# Emerging Technologies and Platforms for the Immunodetection of Multiple Biochemical Markers in Osteoarthritis Research and Therapy

**DOI:** 10.3389/fmed.2020.572977

**Published:** 2020-10-21

**Authors:** Eiva Bernotiene, Edvardas Bagdonas, Gailute Kirdaite, Paulius Bernotas, Ursule Kalvaityte, Ilona Uzieliene, Christian S. Thudium, Heidi Hannula, Gabriela S. Lorite, Mona Dvir-Ginzberg, Ali Guermazi, Ali Mobasheri

**Affiliations:** ^1^Department of Regenerative Medicine, State Research Institute Centre for Innovative Medicine, Vilnius, Lithuania; ^2^Department of Experimental, Preventive and Clinical Medicine, State Research Institute Centre for Innovative Medicine, Vilnius, Lithuania; ^3^Immunoscience, Nordic Bioscience A/S, Herlev, Denmark; ^4^Microelectronics Research Unit, Faculty of Information Technology and Electrical Engineering, University of Oulu, Oulu, Finland; ^5^Laboratory of Cartilage Biology, Institute of Dental Sciences, Hebrew University of Jerusalem, Jerusalem, Israel; ^6^Department of Radiology, Veterans Affairs Boston Healthcare System, Boston University School of Medicine, Boston, MA, United States; ^7^Research Unit of Medical Imaging, Physics and Technology, Faculty of Medicine, University of Oulu, Oulu, Finland; ^8^Departments of Orthopedics, Rheumatology and Clinical Immunology, University Medical Center Utrecht, Utrecht, Netherlands; ^9^Centre for Sport, Exercise and Osteoarthritis Versus Arthritis, Queen's Medical Centre, Nottingham, United Kingdom

**Keywords:** osteoarthritis (OA), biochemical marker, multiplexing technologies, biosensors, nanotechnology, immunodetection, magnetic resonance imaging (MRI)

## Abstract

Biomarkers, especially biochemical markers, are important in osteoarthritis (OA) research, clinical trials, and drug development and have potential for more extensive use in therapeutic monitoring. However, they have not yet had any significant impact on disease diagnosis and follow-up in a clinical context. Nevertheless, the development of immunoassays for the detection and measurement of biochemical markers in OA research and therapy is an active area of research and development. The evaluation of biochemical markers representing low-grade inflammation or extracellular matrix turnover may permit OA prognosis and expedite the development of personalized treatment tailored to fit particular disease severities. However, currently detection methods have failed to overcome specific hurdles such as low biochemical marker concentrations, patient-specific variation, and limited utility of single biochemical markers for definitive characterization of disease status. These challenges require new and innovative approaches for development of detection and quantification systems that incorporate clinically relevant biochemical marker panels. Emerging platforms and technologies that are already on the way to implementation in routine diagnostics and monitoring of other diseases could potentially serve as good technological and strategic examples for better assessment of OA. State-of-the-art technologies such as advanced multiplex assays, enhanced immunoassays, and biosensors ensure simultaneous screening of a range of biochemical marker targets, the expansion of detection limits, low costs, and rapid analysis. This paper explores the implementation of such technologies in OA research and therapy. Application of novel immunoassay-based technologies may shed light on poorly understood mechanisms in disease pathogenesis and lead to the development of clinically relevant biochemical marker panels. More sensitive and specific biochemical marker immunodetection will complement imaging biomarkers and ensure evidence-based comparisons of intervention efficacy. We discuss the challenges hindering the development, testing, and implementation of new OA biochemical marker assays utilizing emerging multiplexing technologies and biosensors.

## Introduction

Osteoarthritis (OA) is the most common form of joint disease and a major cause of pain and chronic disability in older individuals (1). Although OA is primarily associated with aging in the human population (2), there are other key contributing risk factors, including obesity, gender, a history of joint trauma or repetitive use, genetics, heritable metabolic disorders, muscle weakness, underlying anatomical and orthopedic disorders (i.e., congenital hip dislocation), previous joint infection, crystal deposition (i.e., gout), previous rheumatoid arthritis (RA), and various disorders of bone turnover and blood clotting (3).

Biomarkers have been defined in the literature with considerable overlap (4). In general, biomarkers are any quantifiable measurement that can be objectively assessed as an indicator of a biological process, including anatomic, physiologic, biochemical, or molecular parameters. These markers can be associated not only with the presence and severity of specific diseases but also the effects of medical treatments and interventions. The use of laboratory-based biochemical markers in clinical settings is relatively new, and the best strategies to this application are still being developed at medical research level and as well at technological level (i.e., development of reliable detection methods).

Currently, there are no reliable, quantifiable, and easily measured biochemical markers capable of providing an earlier diagnosis of OA, inform on the prognosis of OA disease, and monitor responses to emerging therapeutic modalities (5). The evaluation of structural changes in articular damage via imaging biomarkers [as determined by radiograph or magnetic resonance imaging (MRI)] is the most frequently used in clinical trials to evaluate subject eligibility, and/or efficacy of intervention, supporting decision making in OA drug development by ascertaining treatment effects on joint structure (6). The European League Against Rheumatism (EULAR) has formulated a set of guidelines for imaging applications for the clinical management of OA in peripheral joints (7). However, radiography seems inadequate in the case of OA, as the utility for objective clinical picture evaluation is limited, and in most of the cases, there is no correlation between radiographical and clinical features, especially during early OA stages. This is an example of how radiographic imaging has hampered OA research endeavors (8, 9). MRI thanks to its high sensitivity in showing all involved joint tissues that are clinically relevant at a much earlier disease stage is gaining greater recognition. The importance of MRI in OA diagnosis and prognosis is increasingly emphasized (10–12). However, early detection of structural changes in the joint by MRI does not necessarily serve as an indicator of the existence of clinically defined OA, especially in the absence of symptoms. The potential outcome domains to assess for early OA include patient reported outcomes, features of clinical examination, objective measures of physical function and pain, levels of physical activity, imaging, and biochemical markers in body fluids.

In addition to imaging biomarkers, biochemical markers of joint tissue turnover have the capacity to reflect disease-relevant biological activity and provide useful diagnostic and therapeutic information, enabling a more rational and personalized approach to healthcare management (13). Expert consensus groups have proposed a generally accepted classification of OA biochemical markers according to the disease pathogenesis (14, 15). They include markers of cartilage, bone, and synovial metabolism, which comprise several collagenous proteins, their epitopes and cleavage peptides, various enzymes and non-collagenous proteins, as well as markers of low-grade inflammation: cytokines, chemokines, lipid mediators, and other biochemicals (15). The ratio between the synthesis and breakdown of both articular cartilage and bone can provide an insight into the underlying pathological processes involved in OA, albeit this process may be detected only in late stages of disease pathogenesis. The importance of inflammatory response in OA has also been increasingly highlighted during the past decade, shifting the focus of research investigations on the correlation between biochemical markers of “low-grade inflammation” and disease progression, especially in the context of emerging inflammatory endotypes and phenotypes of OA (16).

The complex evaluation of biochemical markers of extracellular matrix (ECM) turnover, low-grade inflammation, and other biological processes may lead to more specific evaluation of the catabolic and inflammatory aspects of OA. However, most of the studies carried out with biochemical markers to date have focused on late stages of the disease in humans or animal models. Studies in early stages of OA are rare due to the lack of biochemical markers that permit detection of early OA stages.

Early OA refers to the earliest disease stage characterized by emerging clinical symptoms. Early OA does not have a mutually agreed-upon definition, but a group of specialists have proposed a draft classification based on the existence of patient-reported symptoms, clinical examination findings, and minimal to no radiographic signs (17). The definition of early OA is still evolving, and there are ongoing efforts to move toward a consensus definition. Although there is no consensus on an internationally accepted classification, it is generally agreed that identification of the early stages would enable the development of new therapeutics, allowing more targeted and efficacious clinical intervention. However, this requires establishing a clear link between molecular changes in the preradiographic stages, before the onset of clinical and radiographic manifestation of OA (18). Biomarkers indicative of early disease processes and the criteria by which OA is stratified into “early clinical” and “early OA” stages require validation by extensive longitudinal studies that take into account different risk factors, patient subgroups, and types of affected joints (18).

At the present time, none of the currently available biochemical markers are sufficiently capable of discriminating OA diagnosis or prognosis in individuals or provide a consistent outcome measure in OA clinical trials (19). The need for stratification of distinct OA subtypes (the so-called clinical phenotypes) has been recently highlighted (20). This relates to the need for gaining a better understanding of the pathogenesis of OA, the identification of molecular endotypes, and the prospect of developing personalized treatments. Hence, stratifying OA biochemical markers for the detection of molecular endotypes and the enhanced definition of the clinical phenotypes will inevitably expedite the development of personalized OA treatment approaches, as proposed recently (11, 21, 22).

The presence of inflammation has been recognized as a leading component of different OA subtypes (23). Contrast-enhanced MRI of pre-radiographic OA joints revealed that presence of moderate synovitis is very common in knees with clinical OA and is also commonly found in joints without exhibiting concomitant joint effusion (24). Systemic inflammatory mediators, adipokines, released by the adipose tissue, are also involved in the pathogenesis of OA (25), which suggests the convergence, overlap, and interaction of these phenotypes, especially in older individuals with multiple comorbidities.

The inability to assess changes in the joint in the early stages of OA is a major obstacle to making further progress in OA diagnosis and prognosis. Moreover, this stagnancy in biochemical marker development is hampering drug development and thwarting efforts to identify the disease early and mitigate its huge socioeconomic impacts. Therefore, there is an acute need to establish biochemical marker panels or methods for efficient detection to facilitate earlier diagnosis of OA, inform OA prognosis, and monitor therapeutic efficacy of the disease (16, 26). The development of biochemical marker immunoassays has been an ongoing area of activity in OA research and clinical development, but it is still far from being incorporated into a unified framework for disease characterization.

The present article reviews the relevant literature and outlines the main challenges faced by the scientific, medical, and engineering communities in the establishment of the relationship between biochemical markers and OA, as well as the development and design of emerging immunodetection methods such as multiplexing technologies, biosensors, and nanotechnology platforms.

## Biochemical Markers for OA Diagnosis and Prognosis

The currently accepted classification criteria for OA biochemical markers developed by the OA Biomarkers Network has assigned biomarkers into five categories, including burden of disease, investigative, prognostic, efficacy of intervention, diagnostic, and safety (BIPEDs) (27, 28). This categorization provides a framework to describe the potential application of biochemical markers as tools for the early identification of the disease, differentiation of patients based on the extent of disease severity, pre-symptomatic identification of individuals with the disease, or a clinical endpoint used to determine the efficacy of treatment. Biochemical marker detection in serum or urine samples is the least invasive procedure, therefore standing in the first line of clinical interest, while the synovial fluid (SF) biochemical markers are expected to be more reflecting local processes in the joint but have gained more traction in recent years with the increased developmental focus on intra-articular therapies. Although a series of different biochemical markers were assessed for their potential utility, only minimal success was achieved in their clinical validation, highlighting the need for new biochemical markers, representative of joint tissue damage or even linked to particular joints (22, 29). Nevertheless, the major challenge for OA drug development remains the lack of biochemical markers indicating the efficacy of treatment and OA progression (30).

The establishment of normal range intervals of well-phenotyped age-matched controls for 18 separate biochemical markers that were used by the Foundation for the National Institutes of Health/Osteoarthritis Research Society International (FNIH/OARSI) consortium project was an important step in the development of biochemical marker for the diagnosis of OA (31). However, even though some of these biochemical markers showed different distributions between OA subjects and non-OA controls, the overlap of concentration values remains a problem for diagnostic and prognostic applications, as well as the differences between reference intervals based on race, gender, and age, which might necessitate further research into factors that might affect biochemical marker concentrations (31). The variables in baseline levels between individuals pose a significant challenge for biochemical marker development, especially since OA pathogenesis does not embody a singular etiological trajectory, as previously indicated (i.e., inflammatory, bone, metabolic, or age).

## Biochemical Markers of Cartilage Turnover in OA

**Figure 1:**
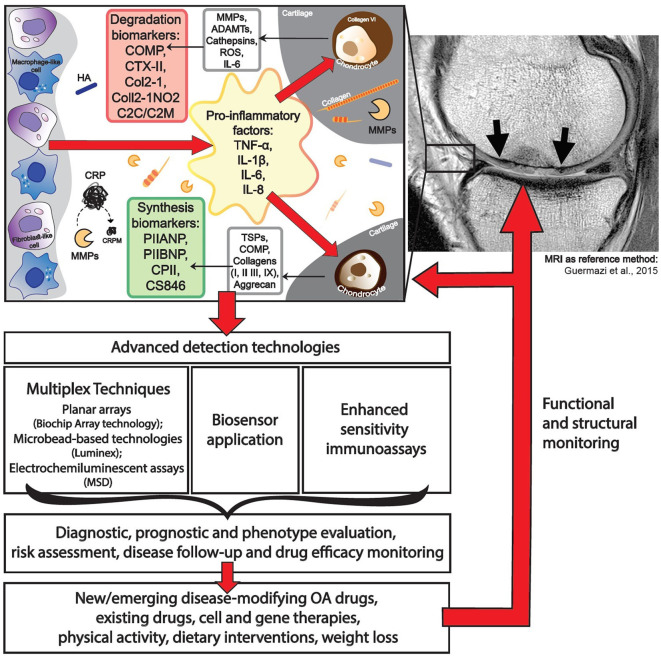
Application of advanced technologies for immunodetection of osteoarthritis biomarkers.

Most OA biochemical markers are dedicated to characterizing cartilage turnover. The most commonly investigated biochemical markers includes the following: ECM degradation—CTX-II, Coll2-1, C2C, C2M, Coll2-1NO2, cartilage oligomeric matrix protein (COMP), aggrecan epitopes (ARGS, TEGE, FFGV), fibulin-3 epitopes (Fib3-1, Fib3-2, Fib3-3), etc.; ECM synthesis—PIIANP, PIIBNP, CPII, CS846, and many others (32) (Figure 1). Some of those cartilage metabolism biochemical markers have gained recognition in the field. For instance, urinary CTX-II (C-telopeptide fragments of type II collagen) is one of the better-known OA biochemical markers that has achieved a superior predictive profile when compared to others (33). Both urine and SF CTX-II were found to be associated with radiographic severity (34), while urine CTX-II was associated with pain in patients with early OA (35).

Another potential biochemical marker widely reported in the OA literature is COMP. Serum COMP is a biochemical marker that has been used in numerous studies because of its ability to distinguish between healthy subjects and OA patients. Furthermore, in the case of COMP, possible prognostic capabilities have been observed (36). Although some studies reported conflicting results, a meta-analysis showed that these biochemical markers (i.e., COMP and CTX-II) could be effective for OA diagnosis and prognosis of progression and differentiation between healthy groups and individuals affected with OA (37). Variation in COMP levels seems more pronounced in SF samples, as compared to serum levels (36). In addition, levels of COMP in SF showed a strong correlation with OA severity, while only a weak inverse correlation to serum COMP levels was found, reinforcing that SF samples are strongly reflective of COMP as an OA biochemical marker (36). Levels of COMP in SF, specifically intact forms of the molecule, were found to be higher in OA patients than in those suffering from other articular disorders, like RA, reactive arthritis, or acute trauma (38). As such, intact COMP appears to better associate with slow progressing and chronic joint pathogenesis, rather than acute proteolytic processes that are associated with severe inflammation in other arthritides, which cause COMP degradation and cleavage. This also highlights the fact that neo-epitopes from the same protein could be generated in diverse manners and thus serve as indicators of various pathological mechanisms related to distinct diseases or disease subtypes (39). Such phenomena should be considered when designing OA analysis platforms, as appropriate antibody selection (with respect to targeted regions) could potentially be linked to better pathogenetic profile validation.

## Biochemical Markers of Low-Grade Inflammation in OA

The most recent definition of OA describes it as disease characterized by low-grade inflammation, distinguishing it from other inflammatory joint diseases. Identification of biochemical markers of low-grade inflammation may identify subtypes of OA, thus contributing to the choice of treatment strategies, particularly with non-steroidal anti-inflammatory drugs, which currently remains among the primary options from the scarce choice of clinically relevant therapies for OA. Notable inflammation-related biochemical markers of OA include CRP, tumor necrosis factor-α (TNF-α), IL-6, and IL-1β, recurring in multiple study reports and systematic reviews (15, 22, 29). The concentrations of these biochemical markers are low, especially compared with other inflammatory diseases of the joint, with TNF-α, IL-6, and IL-1β levels detected at picogram-scale quantities (40), making it challenging to detect their fluctuations in the circulation. For example, CRP levels are consistently elevated in OA patients, with modest variations in its levels among various studies (41). CRP levels were shown to differ between OA patients and non-OA controls and correlated with symptoms of OA, such as pain and function loss; however, these were not found to reflect radiographic findings such as joint space narrowing and KL scores (41). Measurement of hsCRP is a routine laboratory test and the gold standard for evaluation of inflammation (15), making it a relatively translational and accessible transition to OA diagnosis. It is important to note that more attention should be given to known confounding factors of CRP such as body mass index (BMI), race, and gender when interpreting data (42–44). Distinct from full-length CRP are fragments derived from CRP, such as CRPM, which are generated by endopeptidases, such as matrix metalloproteinases (MMPs), which are activated during OA. Indeed, CRPM has been evaluated in RA (45), axial spondyloarthritis (46), and OA (47–49) as a marker of inflammation. The Rotterdam cohort study revealed for the first time that CRPM predicts the risk of OA progression independently of the established biochemical markers uCTX-II and COMP (50).

Elevated levels of IL-1β, IL-5, IL-6, IL-10, IL-13, and TNF-α have been observed in the plasma of patients with knee OA, as compared to SF samples showing unchanged levels (27). Authors of the study attribute either the fluctuation in cytokines in the circulation to the systemic nature of OA or the enhanced permeability of the synovial membrane (40). Similarly, serum TNF-α concentrations were shown to be predictive for radiographic knee OA progression (41) and together with IL-6, were indicative of cartilage loss and joint space narrowing (51). On the other hand, increased SF IL-6, IL-8, and TNF-α levels were associated with pain during movement, while only TNF-α correlated with pain at rest (34).

The selection of inflammatory biochemical markers for OA assessment remains challenging and inconsistent and depends on the purpose, hypothesis, and concept of each study. As there is no consensus on the parameters for the evaluation of low-grade inflammatory activity in OA, a combination of inflammatory and cartilage turnover biochemical markers, in addition to imaging biomarkers, may be the best choice to characterize OA (see section Choice of the Reference Method for Biochemical Marker Detection).

## Emerging Exploratory and Investigative Biochemical Markers of OA: From Mice to Large Animal Models of OA

As previously stated, there is a lack of novel biochemical markers to predict OA clinical status. Moreover, fewer biochemical marker candidates enable clinical decision making about the efficacy of various therapies. The current situation requires pairing between investigative knowledge regarding the disease pathogenesis and the utility of this knowledge to develop novel biochemical markers and therapies.

Searching the current literature for the following phrases including “investigative biomarkers” “osteoarthritis,” and “mice” showed 48 results. Among some of the reports, higher serum levels of FGF21 were displayed in an ACL model of mice, which appeared to be markedly higher in adipsin-deficient mice (52). Another interesting therapeutic target is transglutaminase (TG2), which was shown in various preclinical models to mediate chondrocyte hypertrophy and interleukin-1-induced calcification (53–56). As such, TG2 protein levels in synovial fluid protein have been reported to correlate with the histological grade of OA (57). As the activity of TG2 may be altered in non-OA-related synovitis, better characterization of its levels and bioavailability are needed to assess its potential utility as an OA biochemical marker for human subjects (58). MMP3, an enzyme that degrades proteoglycans and collagens, was associated with early structural OA changes in STR/ort mice (59), which are consistent with human studies showing that its levels correlate with joint width narrowing (60). Another catabolic matrix modifier, cathepsin B, was shown to display increased activity in synovial fluid in various studies (61, 62) and could be paired with imaging of the joint using activity-based probes. Mechanistically, cathepsin B is linked with Sirt1 cleavage and inactivation (63), as well as collagen degradation in cartilage ECM (64). Recently, fragments of Sirt1 were shown to be predictive of chondrosenescence and OA severity using an enzyme-linked immunosorbent assay (ELISA)-based method (65), linking the loss of SIRT1 activity in OA cartilage to its emergence in serum. Lipid profiles in plasma are also reported as biochemical markers of pain and cartilage destruction using the DMM mice models (66). In particular, Pousinis and colleagues, reported six lipid species that increased during posttraumatic OA, identified as cholesterol esters-CE(18:2), CE(20:4), CE(22:6), phosphocholine-PC(18:0/18:2), PC(38:7), and sphingomyelins-SM(d34:1) (66). Phospholipid species were also shown to be increased in human OA plasma (67, 68), indicating that these lipid species may undergo aberrant biosynthesis during OA pathogenesis. The study of lipid mediators as potential marker of OA is an important and promising area of research that highlights the potential for using lipidomics in future studies. However, at the present time, there are no standardized low-cost platforms and easy-to-perform assays for identifying a wide-range of lipid biomarkers.

The use of mouse models for biomarker screening is often advantageous over large animals due to its capacity to undergo skeletal maturation or develop OA more rapidly than larger animals. Moreover, the abundance of genetic mouse models for the validation of pathways and biomarkers related to OA pathophysiology make them good cost-efficient models for initial screening. Indeed, there are many novel findings related to biomarker discovery in mouse models as compared to those available in large animals. Conversely, the use of large animals is justified because they are translational models due to their physiological and anatomical biomechanical similarity to load-bearing human joints and is documented mostly in dogs, sheep, and horses, which exhibit naturally occurring OA (69). When assessing dogs undergoing surgical induction of OA, a significant decrease in TIMP-2 in SF and serum is reported, while MMP-2 was elevated in SF during the progression of OA (70). Coll2-1 and Coll2-1 NO_2_ were correlated with OA changes in the canine ACLT model (71), consistent with the decrease in these serum fragments after HA intra-articular administration to OA patients in a separate study (72).

Assessment of spatial changes in serum proinflammatory cytokines and ECM-related biomarkers was reported for a 4-year training period of posttraumatic OA racehorses. While COMP and CTX-II showed an early increase in serum already at year 2 of training, IL-1β, TNF-α, and IL-6 were mostly elevated at the fourth year of training (73). These data are in line with the approximate 4-fold increase reported for lame training horses vs. non-lame training horses in a separate study using ELISA for the detection of COMP neoepitope (74). NMR analysis of synovial fluid from sheep subjected to anterior cruciate ligament (ACL) reconstruction injury vs sham, revealed significant changes in the following metabolites: isobutyrate, glucose, hydroxyproline, asparagine, serine, and uridine (75). Another study examining the synovial fluid of sheep with OA of the temporomandibular joint detected elevated levels of the active MMP2 in pathogenic SF samples (76). Overall, large animals screened for OA biomarkers exhibit increased levels of matrix-degrading enzymes and ECM fragments (MMP2, COMP, and CTXII), associated with early stages of the disease, while cytokines emerge in the circulation at latter stages, which resembles some of the findings reported for human cohorts (77). In summary, changes in biomarkers are more readily apparent in the synovial fluid of large animals compared to that in serum, as is the case in humans.

Altogether, these biochemical markers, and possibly more, discovered primarily in animal models could contribute to our understanding of OA pathogenesis, become potent drug targets, and serve as potential biochemical markers of OA. The capacity to use genetic mice models as affirmation of a biochemical marker is particularly an important approach for validating candidate investigative and exploratory biochemical markers to associate them with OA and its accompanied joint tissue damage.

## Conventional Immunoassays and Emerging Multiplexing Immunodetection-Based Technologies

Disease prediction by biochemical marker analysis is one of the most promising research areas nowadays. By far, the most widely applied method for biochemical marker quantification is ELISA, which are frequently used to quantify antibodies, peptides, proteins, and hormones in the plasma, serum, urea, or supernatants. ELISA is a highly sensitive, specific, and accurate method. However, ELISAs can only measure a single molecule of interest at a time, which is a major challenge for simultaneous quantification of multiple antigens in huge cohorts of patient samples, in terms of both workload and sample amount. The detection of a single biochemical marker in the serum, intracranial fluid, synovial fluid, urine, or other bodily fluids is not a reliable prognostic or diagnostic indicator in the vast majority of diseases. Single analyte assays are widely applied in protein measurements, while running at least a couple of experiments in parallel increases the time consumption and risk for an error to occur, and a larger volume of analytical samples are needed (78, 79).

Analysis of multiple biochemical marker molecules in every patient could potentially shed better light on disease characteristics and status. However, the measurement of multiple parameters in a single precious sample from a patient in a single run has challenged the scientific and medical community and led to the development and design of assays utilizing multiplexing technologies. Multiplex immunoassays have gained traction in recent years and have already been successfully used in diagnostic tests and cohort screening. Although the majority of developed platforms is still under investigation in the preclinical phase, these techniques can lead to a strong technological revolution in the future as reliable and cost-efficient systems for diagnosis, disease prediction, and monitoring. Two basic assay formats have been developed to facilitate simultaneous detection of multiple analytes: planar arrays (i.e., biochip array technology) and bead-based arrays (e.g., Luminex Technology), and the applications of these assays has been extensively reviewed (78, 80).

The basic principles of biochip planar arrays based on ligand-binding assays were first described in the 1980s (81). Since then, the technology has advanced and attracted the attention from biochemical marker research to clinical diagnoses and prognoses. In antibody microarray systems, large amounts of different antibodies are printed on the planar microarray surface where multiplexed affinity reagents are used to detect and quantify proteins in complex with biological samples (82). In addition to the advantage of detecting multiple biochemical markers, antibody microarrays assays are high throughput and highly sensitive by using small sample volumes and delivering fast results (<24 h from sample preparation to data analysis). Despite the remaining challenges associated with multiplex immunoassay platforms, such as cross-reactivity to off-target biochemical markers and their clinical applicability, there are several systems commercially available (83). For instance, the company MesoScale Discovery® (MSD) offers a multiplex immunoassay platform built upon planar array technology utilizing electrochemiluminescence detection (83, 84). The platform is made in a 96-well format with integrated electrodes to deliver an electric impulse to each well and specific antibodies spotted at the bottom. The detection reagent contains electrochemiluminescent labels that bind to the detection antibody and are only activated by an electric charge, eliminating any background interference by non-specific label detection. This kind of detection system was validated and the sensitivity compared favorably to well-validated single-plex ELISAs (84).

Among planar-array-based technologies, bead-based or cytometric bead array (CBA) platform has also been developed by combining ELISA-based technology with flow cytometry. Beads of different sizes or colors are used for those multiplexed immunoassays (85). Initially, the assay was developed for conventional flow cytometers, but the design of a CBA analyzer by Luminex Company (Luminex Corporation, Austin, TX, USA) enabled streamlining of the workflow and data analysis, making it more accessible. Luminex technology has been adopted by many leading bioscience companies, and various biochemical marker panels have been developed. The Luminex xMAP (multianalyte profiling) technology is a bead-based flow cytometric platform for multiplex analysis. It uses magnetic or polystyrene particles, incorporating two fluorophores in 100 different ratios, giving the possibility to detect 100 analytes (86). The technology offers a greater reproducibility, as compared to the planar arrays, and sensitivity comparable to that of ELISA (87, 88).

The development of a multiplex assay requires overcoming many difficulties, including insufficient detection limits and standardization of the biochemical marker panels for diagnosis and treatment monitoring (89). At the research level, non-traditional methods in clinical settings have been considered as novel strategies to enable high sensitivity and simultaneous detection of a multitude of biochemical markers. Proximity ligation assay (PLA) and, more recently, the proximity extension assay (PEA) have been reported as a sensitive and selective immunoassay method for protein quantification using a pair of DNA oligonucleotides linked to antibodies against the target molecule (90). A multiplexed platform containing 96-plex PEA-based immunoassay was developed to achieve simultaneous measurement of 92 biochemical markers related to cancer, and its performance was evaluated in comparison with benchmark bead-based immunoassays (91, 92). Very recently, a digital PLA (dPLA) has been proposed to simultaneously detect Gram-negative and Gram-positive bacterial DNA as well as the inflammatory biochemical markers IL-6 and TNF-α from patient samples (93). A major advantage of this innovative platform is the use of a digital amplification method, which enables the quantification of very small changes in concentration of the biochemical markers (i.e., subfemtomolar resolution for protein targets). As an outcome, those analyses showed that temporal changes in several biochemical markers, rather than the absolute concentrations, are reliable predictors of patient outcomes.

Surface-enhanced Raman scattering (SERS) has also been considered as a potential detection method of multiple biochemical makers. SERS combines nanostructures made of noble metals (e.g., silver and gold) with Raman spectroscopy, providing a dramatic increase in the characteristic molecular fingerprint offered by Raman spectrum (94). Particular attention has been given to SERS immunoassays, which ensure high sensitivity by SERS detection and high specificity from the antigen–antibody binding. Furthermore, the combination of nanotags and characteristic spectrum of the target molecule makes SERS detection a very attractive strategy to achieve multiplexing as recently demonstrated (94, 95). Photon-upconversion nanoparticles have been used to develop a microtiter plate immunoassay capable of detecting PSA at 1.2 pg/ml, which is 10 times more sensitive than commercial ELISA and covers a dynamic range of three orders of magnitude (96). Such technology has the potential to develop into a new generation of digital immunoassays.

A multiplexed protein detection technology resembling an immunodetection method was developed by SomaLogic Company. The SOMAScan assay utilizes nucleic acid ligands with protein-like side chains—aptamers (SOMAmers) (97). The principle resembles antibody–antigen interaction detection, with affinities comparable and often superior to those of antibodies. SOMAmers have distinct advantages for such applications, including selection conditions not tied to *in vivo* immunization, thermal and chemical stability, smaller size, ease of manufacturing, reliable supply, and full control of lot-to-lot variability (98).

Development of the multiplexed detection assays should inevitably focus on the selection of the detection system/technology and reagents that are the key factors in obtaining sensitivity and specificity. For example, four high-sensitivity cytokine multiplex assays on a Luminex or electrochemiluminescence (MSD) platform were evaluated for their ability to detect circulating concentrations of 13 cytokines as well as for laboratory and lot variability (99). The study showed that no single multiplex panel detected all cytokines, and there were highly significant differences between laboratories and/or lots with all kits. The detection of single IL-6 cytokine was assessed by means of four different immunoassays/platforms (100). IL-6 was measurable in all plasma samples by MSD, while 35, 1, and 4.3% of samples were out of range when measured by Luminex assay, Ultrasensitive Luminex assay (Invitrogen), and High-Sensitivity ELISA (R&D), respectively. Again, it emphasizes the importance of the reagents used for the detection, not only the detection platform.

## Application of Multiplexing Technologies in Diagnostics of Various Diseases

This section describes the lessons from research experiences and challenges observed in various diseases applying multiplexing and diverse innovative technologies for biochemical marker identification and analysis. One of the main challenges is the choice of the most relevant biochemical markers for the prediction and monitoring of a disease. For instance, automated and multiplex biochemical marker assay has been developed to reliably distinguish between RA patients and healthy individuals (101). This study included serum samples from 120 patients. The multiplexed assay was considered to be a relevant and specific method to diagnose RA by using a biochemical marker panel with three biochemical markers yielding a sensitivity of 84.2% and a specificity of 93.8% and using four biochemical markers a sensitivity of 59.2% and a specificity of 96.3%. In another study, quantification of 12 biochemical markers was performed, utilizing a multiplexed sandwich immunoassay in three panels for RA diagnosis (102). This study has demonstrated that the 12 individually selected biochemical markers exhibit a high level of precision with minimal cross-reactivity and interference by substances commonly seen in RA patients. Interestingly, among these two multiplex panels, only two biochemical markers, IL-6 and TNF-α, were included in both studies, while analysis of other biochemical markers generated inconsistent results. Despite that, both studies conclude that their methods provide highly reproducible results, are effective, and can even stratify RA patients into clinically relevant subtypes (102, 103).

Additional analytical capacity has been introduced to the RA biochemical marker field when the recently developed multibiomarker disease activity (MBDA) test was validated in a clinical study on RA patients. The MBDA scores (from 1 to 100) were capable of monitoring changes in disease activity over time and effectively discriminating clinical responders from non-responders in diverse RA cohorts (104). This scoring system is most commonly used to assess the response of biological therapy in RA, however with varying success and utility for the process of tapering and the ceasing of disease-modifying anti-rheumatic drugs (DMARDs). In the AMPLE study of the biological agents Abatacept and Adalimumab, the MBDA score did not reflect clinical disease activity (105), while in the *post hoc* analysis of three cohort studies on Rituximab, the same score was confirmed to represent the clinical response to treatment in RA patients (106). Currently, a prospective, a randomized study of the Vectra DA MBDA blood test is under investigation for Food and Drug Administration (FDA) authorization (107) in diverse RA cohorts.

Disease phenotyping in multiple sclerosis (MS) served as another example of biochemical marker selection. Cases of different subtypes of MS were compared by simultaneous analysis of serum IL-1β, IL-6, IL-8, and TNF-α levels via comparison of two commercially available multiplex platforms (i.e., Luminex-xMAP and Meso Scale Discovery) (108). Although the presence of these biochemical markers was detected in all the subtypes of MS, the levels varied. The significant increase in IL-6 and IL-8 in all the MS subtypes was determined, but a significant increase in TNF-α was observed only in one of the subtypes, as compared to the controls. In addition to the biochemical markers that can be used to diagnose MS, this parameter could be included into the biochemical marker panel for specification of the MS subtype.

Protein array chip immunotechnology has been applied in osteoporosis diagnosis as an alternative method for single biomarker concentration evaluations (109). Individual biomarker assays in osteoporosis, similarly to other multimodal diseases, fail to describe such complex diseases. Single biomarker concentration measurement is currently used to evaluate the progression of osteoporosis (OP) as well as the measurement of bone mineral density (BMD) by dual-energy X-ray absorptiometry (110). The same Immunological Multiparameter Chip Technology (IMPACT) platform that has been applied in RA diagnosis (101) is used to simultaneously measure OP biomarkers CTX-I, procollagen type I N-terminal propeptide (PINP), osteocalcin, and intact parathyroid hormone (PTH). The choice to measure these specific biomarkers was made due to their high sensitivity and suitability to evaluate bone resorption and formation changes. Although the results demonstrated similar analytical performance characteristics to single biomarker assays with an increased sensitivity, the necessity for larger numbers of OP patients as well as inclusion of more biomarkers associated with bone metabolism change indicators in the panel is further needed (109, 111).

Biochemical marker analysis is also becoming a crucial step in cancer diagnostic and predictive/prognostic characterization of the disease stages. The 96-plex PEA immunoassay has been developed and shown to be both sensitive and specific, as well as more scalable, in comparison to traditional immunoassays (91). Such PEA immunoassay has been applied for multiplex analysis of patients with colorectal cancer, which determined the significant correlation of the expression of CEA, IL-8, and prolactin with specific colorectal cancer stage (92). The identical set of 1 μl of plasma samples from patients with colorectal cancer or unaffected controls was run for both assays. Similar quantitative expression patterns were determined for 13 plasma antigens common to both platforms, while the potential efficacy of proximity extension assay was endorsed, as it only demonstrated that the expression of CEA, IL-8, and prolactin are significantly correlated with colorectal cancer stage. Later, another PEA platform for an expanded panel of 275 biochemical markers has been developed and produced a 12 biochemical marker signature algorithm that was comparable to a clinically approved blood-based screening test (112).

Antibody array systems have also been applied in cancer biomarker screening, where simultaneous detection of multiple breast cancer and ovarian cancer biochemical markers, relevant to clinical diagnosis was achieved (113, 114).

It is evident that biochemical marker research in cancer is highly advanced compared to other disease areas, and these studies may serve as good examples of simplified ways for sensitive and specific detection of different cancer types, for instance breast, colorectal, etc. Such state-of-the art technologies as multiplexing a combination of biochemical markers and implementation of biosensors save time and resources for the prediction of treatment response. Other kits mostly cover single-antigen protocols that have been implemented in most clinical laboratories. Cancer biochemical markers profile a panel of different cancer subtypes, where a single biochemical marker might indicate a particular subtype of a disease. However, only 15–20% of patients develop a response to biochemical markers of different cancer subtypes (115). Therefore, larger numbers of biochemical markers can be included, more sensitive and specific diagnostic tool can be developed, and there will be a higher likelihood of positive responses for at least one of the biochemical markers (116). Taken together, the application of multiplex biochemical marker technologies in other disease areas may offer insights that could be implemented as a framework for clinically important OA biochemical marker combination research.

## Multiplexing Advances in OA Research

Due to the capability of measuring up to 100 analytes in one relatively small sample, many different companies have utilized the Luminex xMAP technology platform and created different panels of multiplex assays. Although only a few studies applied this or other multiplex technologies in screening samples from OA patients so far, they generated important data on disease pathogenesis and progression. These data are summarized in Table 1.

**Table 1:** Multiplex assays-based studies in OA.

**Assay**	**Platform**	**Samples tested**	**Detected valuable markers in OA (correlations, associations etc.)**	**References**
Milliplex MAP human cytokine/chemokine panel (42 analytes)	Luminex	Serum and synovial fluid, hip and knee	IL-6, MDC and IP-10 correlated with hip OA. IL-6, MDC, and IP10 were associated with pain in the hip cohort. MCP-1 and MIP-1β were highly expressed in the knee OA	(117)
LINCOplex™ Immunoassay (21 analytes)	Luminex	Knee synovial fluid	MCP-1, MIP-1, IL-2, IL-5 elevated in advanced OA (ICRS scale)	(118)
Pro-human cytokine multiplex assay (33 analytes)	Luminex	Knee synovial fluid	IL-10, IL-12, IL-13, SCGF-β, VEGF correlated with knee pain and function. IL-6, IL-8, IFN-γ, SCGF-β, VEGF, CXCL1 were associated with OA severity (KL scoring)	(119)
Human Luminex Screening Assay (10 analytes)	Luminex	Knee synovial protein extracts	VEGF was decreased in symptomatic OA vs. asymptomatic OA patients' samples. MMP-1 protein increased in OA vs. postmortem controls	(30)
Myriad Human InflammationMAP® 1.0 multiplex immunoassay (47 analytes)	Luminex	Synovial fluid	VEGF, MMP-3, TIMP-1, sICAM-1, sVCAM-1, MCP-1 related to synovial inflammation in OA, radiographic and symptom severity	(47)
BioLegend LEGENDplex human adipokine flow cytometry-based ELISA (13 analytes)	Flow cytometer	Knee synovium cells (24 h cultures *in vitro*/supernatants)	IL-6 expression was highest in mesenchymal cells vs. hemopoietic. One of the patient-specific inflammatory clusters identified had high tissue and mesenchymal cell IL-6 and IL-8 release	(120)
SPRi multiplex assay (9 analytes)	IBIS MX96	Serum and synovial fluid	Early OA markers—IL-1β, IL-6, TNF-α, IFN-γ, IL-10, CCL2, IL-8, IL-4, and C3F high sensitivity (low pg/ml) detection system. Undergoing validation in patient cohort	(121)
Microfluidic FMGC (2 analytes)	Microfluidics	Serum and urine	Simultaneous detection of sCTX-II and uCTX-II. 24-fold and 3.5-fold shorter completion time than the ELISA for urinary and serum CTX-II	(122)

*IL-1β, 2, 4, 5, 6, 8, 10, 12, 13, interleukin 1β, 2, 4, 5, 6, 8, 10, 12, 13; MDC, macrophage-derived chemokine; IP-10, interferon gamma-induced protein 10; MCP-1, monocyte chemoattractant protein-1; MIP-1, 1β, macrophage inflammatory protein 1, 1β; ICRS, International Cartilage Repair Society; SCGF-β, stem cell growth factor β; TIMP-1, metallopeptidase inhibitor 1; sICAM-1, soluble intercellular adhesion molecule-1; sVCAM-1, soluble vascular cell adhesion molecule-1; VEGF, vascular endothelial growth factor; IFN-γ, interferon gamma; CXCL1, C–X–C motif chemokine ligand 1; KL score, Kellgren and Lawrence score; MMP-1, 3, matrix metallopeptidase 1, 3; TNF-α, tumor necrosis factor-α; CCL2, C–C motif chemokine ligand 2; C3F, complement C3 peptide fragment; FMGC, microfluidic fluoro-microbeads guiding chip; sCTX-II and uCTX-II, serum/urinary C-telopeptide fragments of type II collagen*.

A cytokine/chemokine panel was measured in serum from patients with hip and knee OA and compared with that in healthy controls using Luminex platform (117). Endothelial growth factor (EGF), FGF2, MCP-3, MIP-1α, and IL-8 were differentially expressed between hip and knee OA cohorts. In the knee OA samples, EGF was undetectable while MCP-1 and MIP-1β were highly expressed compared with that in hip OA and control samples, suggesting specific differences that may be related to differential disease processes within a given joint. Thus, different inflammatory biochemical marker combinations may represent OA lesions of different joints. These data support findings from an earlier pilot study in human knee synovial fluid (118), showing that among 21 cytokines screened, elevated MCP-1 and MIP-1 in SF were also increased in subjects with advanced arthritis, based on the International Cartilage Repair Society (ICRS) criteria.

Recently, five of the biochemical markers examined in synovial fluid, using cytokine multiplex assay (Luminex), significantly correlated with both knee pain and function (119) (Table 1). Furthermore, significant associations between OA radiographic severity (KL scoring) and some molecules in the synovial fluid were observed. Another 10-plex Luminex assay was applied not on SF but on synovial protein extracts of OA patients undergoing knee replacement surgery (30). Noteworthy, among the proteins analyzed, vascular endothelial growth factor (VEGF) was decreased in the synovium of symptomatic compared with asymptomatic OA samples, which is in contrast to the results of previously mentioned study on synovial fluid (117). Additionally, MMP-1 protein expression was increased in OA compared to postmortem synovial tissue controls.

The importance of evaluating SF biochemical markers as indicators of a symptomatic inflammatory OA endotype has been highlighted in a recent study on 25 patients with radiographic knee OA (47). Levels of 47 different cytokines, chemokines, and growth factors related to inflammation were screened using multiplex immunoassay (Luminex technology). A subset of six SF biochemical markers (Table 1) was associated with synovial inflammation, as well as radiographic and clinical severity, in OA. These six OA-related SF biochemical markers were specifically linked to indicators of activated macrophages and neutrophils. These results attest to an inflammatory OA endotype that may serve as the basis for therapeutic targeting of a subset of individuals at high risk for knee OA progression.

A first detailed comparison between Luminex and MSD multiplex platforms for the analysis of real clinical SF samples from end-stage knee OA was performed on inflammatory cytokines IL-1β, TNF-α, IL-6, and IL-8 (123). Both systems were capable of detecting the selected cytokines, while the MSD platform had a significantly lower limit of detection (LOD) for all four analytes. The authors concluded that MSD platform was better able to detect and quantify low-level analytes (IL-1β and TNF-α) in OA SF samples compared to Luminex, but due to the differences in the antibody pairs and their affinities, such comparisons of technologies are not very conclusive. Noteworthy, the cytokine measurements in OA samples were at best semi-quantitative and depended on the applied platform, assay, and its manufacturer, thus making the comparisons between the technologies complicated.

Flow cytometry and multiplex flow cytometry-based ELISA were employed for the analysis of cell composition and soluble protein production in synovium collected from OA patients undergoing knee replacement surgery (120). Here, IL-6 expression was highest in mesenchymal cells, although in a handful of patients, hematopoietic immune cell (mainly macrophage) expression was more dominant. Using a novel approach, patient-specific inflammatory clusters were identified: they broadly separated into T cell/lymphocyte (1) and myeloid (2 and 3) clusters, with cluster 3, in particular, associated with high tissue and mesenchymal cell IL-6 and IL-8 release. There are preliminary suggestions that these clusters reflect different patient phenotypes, with cluster 2 trending with female sex and cluster 3 with a history of prior joint surgery (arthroscopy/arthroplasty). It remains to be determined if these clusters can be better defined and how they are related to disease progression and clinical phenotypes.

A highly sensitive multiplex assay based on surface plasmon resonance imaging (SPRi) was first developed for the analysis of four cytokines in synovial fluid (IL-1β, IL-6, IFN-α, and TNF-α) (124). Later, by adding several early OA biochemical markers, including complement C3 peptide fragment (C3F), the assay was applied to detect early OA (121). Technologically, specific capture antibodies were spotted on a gold sensor and loaded into the SPRi machine (IBIS MX96). A sample with biochemical markers reached the sensor through a flow cell, and the interactions with the antibodies were measured in real time. The signal was enhanced by adding biotinylated detection antibodies, followed by neutravidin and biotinylated gold nanoparticles, resulting in a signal improvement of over 200 times and an increased sensitivity of more than 10,000 times. The assay is currently undergoing validation in a small patient cohort.

There is a growing evidence supporting the importance of biochemical markers reflecting metabolic changes in cartilage and bone during OA. The panel of metabolic products of cartilage and bone ECM molecules, representing the processes of breakdown (catabolism) or synthesis (anabolism), has been extensively reviewed (28, 32, 33). The prognostic value of the peptides arising from molecular breakdown or synthesis of cartilage ECM is still under investigation. An attempt to multiplex such markers via sandwich and competition immunoassays has been made (122). The new strategy aimed to simultaneously detect the C-telopeptide fragments of type II collagen (CTX-II), which has heterogeneous epitope structure in serum (sCTX-II; homodimers) and urine (uCTX-II; monomers or variant monomers). For the detection of both serum and urinary CTX-II peptides, a microfluidic fluoro-microbeads guiding chip (FMGC) was developed. It has one inlet for sample insertion and four separate chambers, two of which are dedicated for the sandwich-based detection, while the other two were for the competitive immunoassay. The proposed FMGC-based multiple sensing system accurately detected CTX-II, and the results obtained using this assay correlated well with those obtained using commercial ELISA kits. A combination of inflammatory cytokines/chemokines/MMPs together with cartilage/bone synthesis/degradation markers in a single multiplex assay would provide a powerful tool for the classification of OA subtypes and individualized OA treatment strategies.

Taken together, although several novel multiplexing technologies have been tested for biochemical markers associated with OA, the number of such studies remain very limited. The ability to measure the inflammatory cytokines in a multiplexed manner is not yet translated to the combined multiplex assays for cartilage/bone metabolic markers and not well-controlled with a sensitive reference method as MRI. These data could lead to understanding the role and importance of inflammation in the processes of cartilage breakdown and regeneration and specify the need for intervention. Moreover, most of the multiplexed OA biochemical marker studies to date were performed on SF samples, while only few studies involved the analyses of serum or urine, which would arguably better serve the major clinical need for OA monitoring.

## Nanotechnology Strategies to Enhance Selectivity and Sensitivity of Biochemical Marker Detection

The major drawback, particularly related to serum or urinary biochemical markers, is that some of them are found at lower than picomolar concentrations, which are too low to be detected by conventional methods such as ELISA. Therefore, the analytical techniques, offering high degrees of sensitivity and specificity, such as those employing nanomaterials, proximity ligation, or digital platforms (e.g., digital ELISA), might appear useful for the analysis of ultralow concentrations of biochemical markers in a clinical setting (125). The recent advances in material science, nanotechnology, and bioconjugation techniques have enabled the application of a large diversity of nanomaterials to enhance the sensitivity of advanced immunoassays (126). Only a few studies have been reported about enhanced immunoassays specifically designed for OA biochemical markers (Table 2). For instance, a quantitative lateral-flow immunoassay technique with antibodies conjugated to gold nanoparticles was used for the detection of COMP in the synovial fluid. The proposed method showed similar results to corresponding ELISA, with an average difference of <7% without the need of expensive equipment or complex procedures (127). However, most of the reported studies have investigated the use of advanced nanomaterial-enhanced immunoassays toward inflammatory biochemical markers.

**Table 2:** Enhanced immunoassays for detection of biochemical markers relevant to OA.

**Method**	**Samples tested**	**Biomarker**	**Advantages**	**Characteristics**	**References**
Quantitative lateral flow immunoassay using antibody-conjugated gold nanoparticles	OA patient SF	COMP	Cost effectiveness	Dynamic detection range: 0.6–20 μg/mL	(127)
Quantum dot-linked immunosorbent assay (with immobilized orientation-directed half-part antibodies)	Antigen solution	IL-6	High sensitivity	Lower LOD: 50 pg/Ml	(128)
SENSIA	Pooled human serum	15-Plex (including IL-1β, IL-2, IL-4, IL-6, IL-10, MMP-9, TNF-α)	Cost effectiveness	IL-1β, IL-2, IL-6, and 2 other markers were in good agreement with FLISA (>0.9R^2^)	(129)
Surface-enhanced Raman scattering based immunoassay	Healthy volunteer blood samples	IL-6, IL-8, and IL-18	High sensitivity	LOD: IL-6, 3.8 pg/ml; IL-8, 7.5 pg/ml; and IL-18, 5.2 pg/ml	(130)
Electrochemiluminescence-based system	Serum samples	CRP	High sensitivity, good selectivity, good reproducibility, and low cost	Range: 0.05–6.25 ng	(131)
Microfluidic immunoassay with streptavidin–biotin–peroxidase nanocomplex	Unspecified patient serum samples	IL-6 (multiplexed with procalcitonin)	High sensitivity	Detection range, 5–1,280 pg/ml; LOD, 1.0 pg/ml	(132)
Combined electrochemiluminescent and electrochemical immunoassay	Serum samples	IL-6	Broad dynamic range, high sensitivity, and selectivity	Detection range, 10 ag/ml−90 ng/ml	(133)

*COMP, cartilage oligomeric matrix protein; IL-1β, 2, 4, 6, 8, 10, 18, interleukin 1β, 2, 4, 6, 8, 10, 18; LOD, limit of detection; SENSIA, silver-enhanced sandwich immunoassay; MMP-9, matrix metalloproteinase 9; TNF-α, tumor necrosis factor-α; FLISA, fluorescence-linked immunosorbent assay; CRP, C-reactive protein*.

Markers of inflammatory activity have been at the forefront of detection limit improvement, as the concentrations of immune analytes are notoriously low, particularly in the serum or urine (134). For instance, an ELISA-like method based on the nanometer-sized fluorescent semiconductor particles called quantum dots has detected concentrations of IL-6 as low as 50 pg/ml (128). Application of silver-enhanced sandwich immunoassay (SENSIA) showed comparable results to the fluorophore-linked immunosorbent assay for the detection of IL-6, IL-2, and IL-1β in serum samples while being more cost effective (129). IL-6 and IL-8 showed improved detection limits (2.3 and 6.5 pg/ml, respectively) with sensitive surface-enhanced Raman scattering-based immunoassays in comparison to ELISA counterparts (130).

Electrochemiluminescence-based systems have also been applied for the analysis of inflammatory markers. Application of such label-free electrochemiluminescent immunosensor that utilizes the poor conductivity of CRP molecules bound to antibodies has enabled its detection at the limit of 0.011 ng/ml (131). An immunoassay based on mesocrystal nanoarchitectures combined with electrochemiluminescent and electrochemical detection has been presented to quantify IL-6 (133). The results showed high sensitivity by achieving a broad linear dynamic range of 10 ag/ml−90 ng/ml.

A quantitative microfluidic immunoassay combined with the streptavidin–biotin–peroxidase (SA-B-HRP) nanocomplex-signal amplification system (MIS) has also been presented to detect IL-6 simultaneously to a second inflammatory biochemical markers (i.e., procalcitonin). In this case, the linear range for IL-6 detections was 5–1,280 pg/ml, and the limit of detection was 1.0 pg/mL, which was significantly improved compared with microfluidic immunoassays without amplification systems. Despite these promising outcomes toward inflammatory biochemical markers, to our knowledge, the enhanced-detection immunoassays mentioned above have not yet been applied toward OA inflammatory response.

## Novel Strategies to Enable Diagnostic and Prognostic Monitoring Via Biosensors

Biochemical markers have the potential to be used as indicators of changes in the course of the disease, which might signal the need for additional imaging tests or changes in the course of OA, which could require treatment option re-evaluation (11). However, as a monitoring tool, they will only be considered if there are methods available to easily detect the subtle changes in concentration values while maintaining consistency in data collection. A potential solution to this is the application of biosensors, which are analytical devices that analyze biological responses and convert them into measurable physicochemical signals, typically exhibiting high specificity and reusability (135). Biosensors are an expanding field toward rapid, easy, and reliable detection of relevant biochemical marker that have been implemented in the diagnosis of various diseases and have also started to be considered as potential tools for OA research and clinical application (Table 3).

**Table 3:** Biosensors for detection of biochemical markers relevant to OA.

**Method**	**Samples tested**	**Biochemical marker**	**Advantages**	**Characteristics**	**References**
Quartz crystal microbalance biosensor	Urine of OA patients and healthy controls^*^	COMP	Reaction time advantage, high sensitivity	Detection range: 1–200 ng/ml	(136)
Nanoparticle amplified SPRi aptasensor	Human serum	CRP	High sensitivity	LOD: 5 fg/ml	(137)
Quartz crystal microbalance biosensor	MMP-1 controls	MMP-1	Reaction time advantage	Detection range: 2–2,000 nM	(138)
Fiber optic-particle plasmon resonance biosensor integrated with microfluidic chip	OA patient SF^*^	MMP-3	Cost-effectiveness, portability, high sensitivity	–	(139)
Fiber-optic particle plasmon resonance biosensor	OA patient SF^*^	TNF-α and MMP-3	Reaction time advantage, simple usage, high sensitivity, high selectivity	LOD: TNF-α, 8.2 pg/ml; MMP-3, 8.2 pg/ml	(140)
Fluoromicrobeads guiding chip-based system	Human SF and serum^*^	COMP	Reaction time advantage	Detection range: 4 and 128 ng/ml	(141)
Fluoromicrobeads guiding chip-based system	Human urine-based controls and artificial serum	uCTX-II and sCTX-II	Simultaneous detection, reaction time advantage	Linear detection range: sCTX-II, 0.1–2.0 ng/mL; uCTX-II, 200–2,800 ng/mmol	(122)
Ultraviolet–visible spectroscopy	uCTX-II controls	CTX-II (multiplexed with glucose)	Cost effectiveness and simple manufacturing	Detection range: 1.3–10 ng/ml	(142)
Ambient light-based optical biosensor	uCTX-II epitope controls	uCTX-II	Cost effectiveness, simple usage	LOD: 0.2 ng/ml	(143)

* *Analyzed in OA patient samples*.

*MMP-1, 3, matrix metallopeptidase 1, 3; TNF-α, tumor necrosis factor-α; sCTX-II and uCTX-II, serum/urinary C-telopeptide fragments of type II collagen; LOD, limit of detection; SENSIA, silver-enhanced sandwich immunoassay; COMP, cartilage oligomeric matrix protein; CRP, C-reactive protein*.

COMP has been measured in both OA patient's synovial fluid and serum with an immunosensing device using FMGC technology that has a reaction time advantage over conventional ELISA (141). As it shows good correlation to results obtained by conventional ELISA (coefficient of variation was only within 7%), it could potentially be used in clinical settings, more so if the technology advances to a multiplex format. FMGC-based system has also been used for uCTX-II and sCTX-II quantification, which not only analyzed both factors simultaneously but was also faster than the conventional ELISA by 25- and 3.5-fold, respectively (122). Both systems exhibited high sensitivity and very similar LODs to their respective ELISAs.

Several optics-based biosensing platforms have also been investigated. Low-cost optical biosensing platforms, based on ultraviolet–visible spectroscopy, have been developed for uCTX-II using just common office supplies for data reading (142). As the detection range falls into the clinically relevant intervals, this method of analysis could be introduced into clinical immunoanalysis. The same biochemical marker, uCTX-II, was analyzed using a smartphone-embedded illumination biosensor that had a high accuracy under various lighting conditions (143). Due to its low-cost fabrication requirements and satisfactory detection capabilities in both indoor lighting and in sunlight, it has the potential of being used as a point-of-care diagnostic tool. Real-time multiplexed analysis of MMP-3 and TNF-α was achieved using a single fiber-optic particle plasmon resonance biosensor, which could be used for monitoring both inflammatory and cartilage breakdown activity simultaneously (140). The same type of biosensor has been used for the detection of inflammatory biochemical marker IL-1β with results comparable to a corresponding ELISA method (limit of detection, 21 pg/ml), which, together with the relatively low cost, shorter analysis time, and small sample requirements, seems very attractive for the use in a clinical setting (144).

Other potential candidates for innovative biosensing approaches are technologies that use the piezoelectric effect and transform mechanical stress into quantifiable electrical current. For instance, a quartz crystal microbalance (QCM)-based biosensor for COMP was developed and, compared to ELISA data, showed high accuracy in a shorter analysis time frame (136). Similar biosensor has been demonstrated to detect MMP-1 levels at concentrations between 2 and 2,000 nM in <10 min with a lower detection limit of 2 nM (138). While the clinical relevance of COMP was demonstrated due to its association to the OARSI grades of OA progression, MMP-related biochemical markers might be useful in determining the activity of the processes of cartilage degradation. QCM-based strategies are user friendly and quicker than ELISA, making it a suitable application as a homecare device, comparable to those used for blood glucose monitoring.

Seeking to overcome the problems faced by conventional ELISA method, such as high cost and long process duration, electrochemical-impedance-based immunoassays have been applied to determine levels of the bone-related degradation biochemical marker CTX-I (145).

Advanced biosensors technology has also exploited nanotechnology to enable high sensitivity detection of biochemical markers, potentially leading to applications in clinical settings. Nanoparticle-enhanced plasmonic biosensor have been demonstrated to detect inflammatory marker CRP in only 2 h at concentrations four orders of magnitude lower than the clinically relevant concentrations (146). This ultrasensitive biosensor is fabricated using scalable and low-cost manufacturing, providing a powerful platform for multiplexed biochemical marker detection in several settings. Nanoplasmonic biosensor microarrays have been demonstrated for parallel multiplex immunoassays of six cytokines (i.e., IL-2, IL-4, IL-6, IL-10, TNF-α, IFN-γ) in a complex serum matrix on a single device chip (147). The device was fabricated using easy-to-implement, one-step microfluidic patterning and antibody conjugation of gold nanorods. The proposed biosensor showed the capability to measure cytokine at concentrations down to 5–20 pg/ml from a 1-μl serum sample within 40 min. Electrochemical immunobased biosensors in combination with carbon nanomaterials have also been reported to detect inflammatory biochemical markers (148, 149). Simultaneous detection of IL-1β and TNF-α using human serum and saliva was achieved using dual-screen printed electrodes modified with carbon nanotubes (148). The proposed biosensor showed improved analytical performance with respect to previous approaches and ELISA methods by achieving limits of detection at 0.38 pg/ml (IL-1β) and 0.85 pg/ml (TNF-α), within 2 h and 30 min and significantly less reagents consumptions.

Diverse cost-effective biosensors for OA that minimize the duration of analysis or in other ways outperforming conventional methods are under development, with a subset of them proposed as clinically suitable biochemical marker detection tools or even potential point-of-care monitoring devices. Although there are multiple technological approaches that are currently under investigation to determine the applicability of various OA biochemical markers, studies that sensitively and consistently follow the changes in levels of OA biochemical markers throughout the course of the disease and elucidate diurnal variation, responses to physical activity, anti-inflammatory medication, or nutrition are still a lacking. For instance, despite that large diversity of biosensors have been developed to detect the inflammatory biochemical marker CRP as reviewed in Vashist et al. (150), CRP biosensors were not tested on OA patient serum samples so far. CRP biosensors created have already been designed reaching detection levels in the zeptomolar concentrations (137). Of note, it is likely that monomeric and multimeric forms of CRP may possess different catabolic and inflammatory profiles. Therefore, new assays need to be developed to distinguish between monomeric and multimeric forms of CRP and other proteins. The application of biosensors, ideally for several multiplexed biochemical markers, might lead to new insights into the role of inflammation in the pathological processes of cartilage turnover in OA.

## Choice of the Reference Method for Biochemical Marker Detection

One of the major drawbacks in biochemical marker development for OA is a lack of relevant reference methods to validate their efficacy. Most studies so far have employed radiographic assessment of KL grade for scoring of OA stages, which is neither sensitive, specific, nor easily reproducible in longitudinal clinical trial (10). The lack of a sensitive reference biochemical marker (either imaging or biochemical marker) have likely led to difficulties in proving the utility of biochemical markers in OA. The close correlation between “wet” (biochemical analyte, genomic, etc.) and “dry” (radiography, MRI, or clinical evaluation finding, etc.) as a reference method in each case is of major importance for the successful implementation of biochemical markers (Figure 1). The prospective CHECK study investigates five clusters of biochemical markers, related to specific pathogenic processes: “bone,” “inflammation,” “synovium,” “adipokines,” and “cartilage synthesis,” which will be validated via early radiographic KL scoring of OA status as a reference method (151). Duration of this study is planned for 10 years, and it will finish in 2022.

MRI technology is increasingly implemented as a reference method for evaluation of OA status (152). Several studies involving the analysis of multiple biochemical markers in serum and synovial fluid of knee OA patients (22) use MRI as a reference method for knee damage evaluation. The relation of biochemical serum markers to MRI data in studies performed in 2018 and 2019 are summarized in Table 4.

**Table 4:** Correlation between biochemical marker levels and MRI data.

**Study design, number of patients**	**MRI scoring system**	**Detection method/biochemical marker panel**	**Biochemical markers association with MRI scoring data**	**References**
Case–control (*n* = 600) Follow-up points: baseline, 12 and 24 months	MOAKS: Hoffa synovitis and effusion synovitis	ELISA: HA, MMP-3, Coll2–1NO2	HA and MMP-3 were modestly associated with effusion-synovitis at baseline	(153)
Cross-sectional (*n* = 89)	WORMS	ELISA: COMP, MMP-3, Coll2-1, Turbidimetric analysis: CRP	COMP correlated positively with WORMS and MMP-3. WORMS scoring data are not provided	(154)
Case control (*n* = 141) Follow-up: 2 years	WORMS	ELISA: HA, MMP-3, COMP, Coll2-NO2, uCTX-II, PIIANP, CTXI, CS846, C2C, CPII, NTXI/uNTXI, C12C/uC12C	MRI data associated between HA, COMP, and MMP-3 biochemical markers of OA. The biochemical cartilage ECM (Coll2-NO2) degeneration reflects MRI T2 measures	(155)
Cross-sectional (*n* = 160) Follow-up: 2 years	WORMS	ELISA: IL-8, COMP, CTXI, NTXI, PIIINP, MMP-3, MMP-10, MMP-13	The positive association was between IL-8 and infrapatellar fat pad signal intensity	(156)
Cross-sectional (*n* = 141) Follow-up: 2 years	WORMS	ELISA: 100A8/A9, MMP-3, MMP-10, MMP-13	The levels of alarmins 100A8/A9 had positive associations with MRI score for total and local cartilage defects (lateral femoral, lateral tibial, and medial femoral sites)	(157)
Osteoarthritis Initiative Progression subcohort (*n* = 583) Follow-up: 2 years	MRI quantitative cartilage volume measurement	ELISA: adiponectin LUMINEX: adipsin chemerin, leptin, visfatin, IL-8, MCP-1, CRP	The ratio of adipsin/MCP-1 was associated with the MRI knee structural changes, and CRP/MCP-1 with symptoms in obese OA subjects	(158)
Cross-sectional (*n* = 16) Follow-up: 5 years	MRI semiautomatic segmentation method	ELISA: COMP, C1,2C, CS846	Long-term mechanical stimuli increase the cartilage degradation markers as C1,2C and CS846. Those biochemical markers correlate with cartilage damage (MRI)	(159)
Multicenter, double-blind, phase III clinical trial (*n* = 163) Follow-up: 1 year	WORMS	ELISA: CTX-I, COMP, PIIANP, MMP-3, C1M, C3M, C2M, CS846, CTX-II, uCTX-II/creatinine ratio	Clinical study of cell and gene therapy: no significant differences in MRI between the groups of treatment vs. placebo	(160)
Randomized, double-blinded, sham-controlled trial (*n* = 55) Follow-up: 3 months	MOAKS: Hoffa synovitis and effusion synovitis	ESR ELISA: hsCRP	Low-dose radiation therapy does not induce significant effects on inflammatory signs assessed by MRI, ultrasound and serum inflammatory markers	(161)

*s100A8/A9, myeloid related protein 8/14, calprotectin; ESR, erythrocyte sedimentation rate; COMP, cartilage oligomeric matrix protein; Coll2-1, type II collagen degradation biomarker 1; Coll2–1NO2, nitrated epitope of the α-helical region of type II collagen; C12C/uC12C, collagen type I and II cleavage product and its urine form; C2C, type II collagen degradation biomarker, generated from C-terminus fragment; C1M, C2M, C3M, matrix metalloproteinase (MMP) mediated type I, II, III collagen degradation biomarker; CPII, C-propeptide of type II procollagen; CRP, C-reactive protein; CTX-I, serum C-terminal telopeptide of type I collagen; CS846, chondroitin sulfate synthesis marker; sCTX-II and uCTX-II, serum/urinary C-telopeptide fragments of type II collagen; HA, hyaluronic acid; IL-8, interleukin 8; MMP-3, 10, 13, matrix metalloproteinase 3, 10, 13; MCP-1, monocyte chemoattractant protein-1; MOAKS, MRI OA Knee Score; NTXI/uNTXI, crosslinked N-telopeptide of type I collagen and its urine form; PIIANP, serum N-propeptide of collagen IIA; PIIINP, N-terminal procollagen III propeptide; WORMS, whole organ magnetic resonance imaging score*.

The listed studies revealed associations of different biochemical markers with MRI scoring of synovial inflammation and cartilage degradation. It is important to note that these MRI techniques can detect early bone marrow lesions that may be associated with the onset of cartilage degradation and correlate with inflammation in joint tissue (162). As these data are ambiguous and associated with various aspects of synovial inflammation and/or cartilage damages, no perfect scoring system exists to date. In terms of complexity, heterogeneity, and size of knee OA-related data, it is considered a “big data” issue; therefore, machine learning and application of computer algorithms has attracted significant interest for the evaluation of biochemical and imaging markers (163). A recent publication by Emery et al. described outcome measures for early OA that could be useful in clinical practice and/or the research setting (12). A consensus-based OA phenotype framework was created with intent to facilitate research on OA phenotypes and increase combined efforts to attain effective OA phenotype classification, by providing a number of coherent definitions and statements and a set of reporting recommendations that were supported by a panel of experts in OA research field (164). Many other research groups are currently working with regulatory agencies across the world seeking to clinically qualify confirmed new biochemical markers and imaging biomarkers (165, 166).

## Development of Reference Methods

Looking into the future of radiological reference method development, more attention will be focused on morphological MRI, observing “premorphological” biochemical compositional changes of articular tissues (12, 18). Compositional MRI techniques evaluate cartilage composition [glycosaminoglycans (GAGs), proteoglycans, or collagens] and hydration. Compositional MRI of cartilage matrix changes can be performed using advanced MRI techniques such as delayed gadolinium-enhanced MRI of cartilage (dGEMRIC), T1 rho, and T2 mapping (152). The chemical exchange saturation transfer of GAG (GagCEST) can detect cartilage endogenous GAG content without the need of intravenous contrast injections or special hardware (167). Negatively charged GAGs attract sodium cations and their distribution in sodium MRI correlates with GAG content in cartilage (152). It is consequently important to harness morphological MRI, compositional changes of the cartilage (GAGs, proteoglycans, or collagens), and hydration from images and to establish a correlation between them and quantitative biochemical markers.

In differential diagnosis for OA, it is important to evaluate the intensity of inflammatory component expression, which usually is not as high as in other inflammatory arthritides. The hybrid imaging techniques as the positron emission tomography (PET) method with 18F-fluorodeoxyglucose (FDG) uptake reflected the inflammatory activity, associated with elevated inflammatory cytokine levels, suggesting that FDG-PET may be effective for quantification of the inflammatory activity in different rheumatic diseases (168, 169). The inflammation may contribute to the increased FDG uptake in OA, reflecting the rate of disease progression and inflammatory phenotype. Single-photon emission computed tomography combined with the high-resolution computed tomography (SPECT/CT) technique can visualize folate receptor positive cells, representing activated macrophages and neutrophils, which is associated with the inflammation in the joints of OA patients (47).

These new technological approaches are may be systematically applied for the identification and characterization of OA phenotypes and, together with the relevant biochemical and imaging markers, might contribute to a better understanding of the role of low-grade inflammation in the development of OA, as well as facilitate the identification of the need for anti-inflammatory medication (11). The local inflammatory activity associated with elevated inflammatory cytokine levels (169) and folate receptor positive cells, representing activated macrophages and neutrophils, can already be visualized (47). The rapid progress of such sophisticated imaging methods should lead to the precision in imaging biomarker choice and implementation.

## Conclusions

In this article, we have provided an overview of several technologies, platforms, and strategies that facilitate and improve biochemical marker detection. Application of such novel techniques in imaging and biochemical marker identification may lead to better definition of OA phenotypes and categories and add complementary value to radiologically validated outcome measures in clinical practice and/or the evidence-based comparisons into the effectiveness of therapeutic interventions.

OA biochemical marker immunoassays are potentially viable tools for OA evaluation, monitoring, and drug development. However, specific problems, such as low biochemical marker concentrations, patient-specific variation, limited utility of single biochemical markers to get definitive characterization of OA status, and application of relevant reference methods, require innovative approaches to produce clinically relevant biochemical marker biosensors. Such technologies are already on the way to establishment in routine diagnostics and monitoring of other diseases and could potentially serve as good technological platforms for early OA characterization. Inflammatory markers are monitored in many inflammatory diseases, and novel technologies like biosensors dedicated to reducing costs or time of their detection may also be successfully implemented in OA. Multiplexing inflammatory biochemical markers in combination with biochemical markers of cartilage matrix turnover may shed light on poorly understood mechanisms involved in OA pathogenesis and lead to a better understanding of the role of low-grade inflammation in OA pathogenesis and better classify different clinical phenotypes and molecular endotypes of OA, leading to personalized therapeutic approaches for OA.

## Author Contributions

EBe: conceptualization and review methodology. EBa, GK, PB, IU, and UK: acquisition of resources. EBe, EBa, GK, PB, IU, and UK: writing—original draft. EBe, EBa, GK, PB, IU, UK, MD-G, and AM: writing—revision. EBe, EBa, GK, CT, HH, GL, AG, MD-G, and AM: review and further editing. EBe and AM: supervision. AM: final submission and funding acquisition. All authors contributed to the article and approved the submitted version.

## Conflict of Interest

AG is a shareholder of BICL, LLC and consultant to Pfizer, Galapagos, TissueGene, MerckSerono, AstraZeneca, and Roche. AM has consulted for Abbvie, AlphaSights, Artialis SA, Flexion Therapeutics, Galapagos, Guidepoint Global, IAG, Kolon TissueGene, Pacira Biosciences Inc., Pfizer Consumer Healthcare (PCH), Servier, and Science Branding Communications and received research funding from the European Commission (FP7, IMI, Marie Skłodowska-Curie, ES Strukturines Paramos), Versus Arthritis (Arthritis Research UK), Pfizer Inc., Kolon TissueGene, and Merck KGaA. CT is an employee of Nordic Bioscience. The remaining authors declare that the research was conducted in the absence of any commercial or financial relationships that could be construed as a potential conflict of interest.

## Glossary

ACLT: anterior cruciate ligament transection
ARGS, TEGE, FFGV: Aggrecan epitopes
BIPEDs: burden of disease, investigative, prognostic, efficacy of intervention, diagnostic and safety
C12C/uC12C: collagen type I and II cleavage product and its urine form
C1M, C2M, C3M: matrix metalloproteinase (MMP) mediated types I, II, III collagen degradation biomarker
C2C: type II collagen degradation biomarker, generated from C-terminus fragment
C3F: complement C3 peptide fragment
CA153, CA125: cancer antigen 153, 125
CBA: cytometric bead array
CCL2: C–C motif chemokine ligand 2
CEA: carcinoembryonic antigen
CHECK: Cohort Hip and Cohort Knee
Coll2-1: type II collagen degradation biomarker 1
Coll2-NO2: nitrated form of type II collagen degradation
COMP: cartilage oligomeric matrix protein
CPII: C-propeptide of type II procollagen
CRP: C-reactive protein
CRPM: neo-epitope of MMP-1 and MMP-8 mediated degradation of C-reactive protein
CS846: chondroitin sulfate epitope 846
CXCL1: C–X–C motif chemokine ligand 1
dGEMRIC: delayed gadolinium-enhanced MRI of cartilage
ECM: extracellular matrix
EGF: endothelial growth factor
ELISA: enzyme-linked immunosorbent assay
ESR: erythrocyte sedimentation rate
EULAR: European League Against Rheumatism
FDA: Food and Drug Administration
FDG: 18F-fluorodeoxyglucose
FGF2: fibroblast growth factor 2
Fib3-1,2,3: fibulin-3 epitopes 1,2,3
FLISA: fluorescence-linked immunosorbent assay
FMGC: fluoro-microbeads guiding chip
FNIH: Foundation for the National Institutes of Health
GagCEST: chemical exchange saturation transfer of GAG
GAGs: glycosaminoglycans
HA: hyaluronic acid
hsCRP: high-sensitivity CRP
ICRS: International Cartilage Repair Society
IFN-γ: interferon gamma
IL-1β: 2, 4, 5, 6, 8, 10, 12, 13, interleukin-1β, 2, 4, 5, 6, 8, 10, 12, 13
IP-10: interferon gamma-induced protein-10
KL: Kellgren and Lawrence
LOD: limit of detection
MAP: multianalyte profiling
MCP-1,3: monocyte chemoattractant protein-1,3
MBDA: multibiomarker disease activity
MDC: macrophage-derived chemokine
MIP-1: 1α, 1β, macrophage inflammatory protein-1, 1α, 1β
MMPs: matrix metalloproteinases
MOAKS: MRI OA Knee Score
MRI: magnetic resonance imaging
MS: multiple sclerosis
MSD: Meso Scale Discovery
MSP: methylation-specific PCR
NTXI/uNTXI: crosslinked N-telopeptide of type I collagen and its urine form
OA: osteoarthritis
OARSI: Osteoarthritis Research Society International
OPG: osteoprotegerin
PDGFR: platelet-derived growth factor receptors
PET: positron emission tomography
PIIANP: serum N-propeptide of collagen IIA
PIIBNP: serum N-propeptide of collagen IIB
RA: rheumatoid arthritis
SCGF-β: stem cell growth factor-β
sCTX-II and uCTX-II: serum/urinary C-telopeptide fragments of type II collagen
SENSIA: silver-enhanced sandwich immunoassay
SERS: surface enhanced Raman scattering
SF: synovial fluid
sICAM-1: soluble intercellular adhesion molecule-1
SIRT1: deacetylase sirtuin-1
SPECT/CT: single-photon emission computed tomography combined with high-resolution computed tomography
SPRi: surface plasmon resonance imaging
sVCAM-1: soluble vascular cell adhesion molecule-1
TIMP-1: metallopeptidase inhibitor-1
TNF-α: tumor necrosis factor-α;VEGF, vascular endothelial growth factor
WORMS: whole-organ magnetic resonance imaging score.
